# An Open-Phase Pilot Trial of an Integration of Prolonged Exposure Therapy and the Coping Long-Term With Active Suicide Program

**DOI:** 10.1016/j.cbpra.2025.06.002

**Published:** 2026-05-05

**Authors:** Lily A. Brown, Marin Kautz, Wendy Muzzy, Stephanie Hart, Jonna Vaughn, Sally Murphy, Edna Foa, Ivan Miller, Adam Kahn, Ron Acierno

**Affiliations:** University of Pennsylvania; University of Pennsylvania; Ralph H. Johnson VA Health Care System; Ralph H. Johnson VA Health Care System; Ralph H. Johnson VA Health Care System; Ralph H. Johnson VA Health Care System; University of Pennsylvania; Warren Alpert Medical School of Brown University and Butler Hospital; University of Pennsylvania; Ralph H. Johnson VA Health Care System and University of Texas Health Science Center at Houston

**Keywords:** Posttraumatic stress disorder, Suicide ideation, Prolonged exposure, Coping long-term with active suicide program

## Abstract

Posttraumatic stress disorder (PTSD) is associated with an increased risk for suicidal ideation and behavior, yet clinical practice guidelines for addressing suicide risk in the context of PTSD are not yet available. This open trial evaluated the safety and preliminary efficacy of an integration of a trauma-focused treatment, prolonged exposure therapy (PE), with the coping long-term with an active suicide program (CLASP). CLASP-PE was delivered to patients with PTSD who reported suicidal ideation with intent, a plan, or a past-month attempt. Veterans or active-duty military personnel with a confirmed PTSD diagnosis and suicide risk (N = 14; 86% male) began the CLASP-PE protocol, which was delivered via telemedicine in 10–16 sessions, including a session with a support person as feasible. Two participants only provided baseline data; of the remaining 12, 8 participants (n = 66.7%) completed their treatment sessions. Participants reported significant reductions in PTSD symptoms (p < .05), suicidal ideation (p < .05), and depression symptoms (p < .001). The degree of reduction in PTSD symptoms and depression symptoms in treatment predicted the degree of reduction in suicidal ideation in treatment. No suicide attempts or serious adverse events occurred in treatment. A case vignette is shared. CLASP-PE was safe, with preliminary evidence supporting its efficacy. Additional randomized controlled trial research is needed to evaluate its impact compared to a control group. If replicated, the CLASP-PE protocol would be a useful and safe clinical tool for individuals with PTSD who are experiencing significant suicide risk.

Posttraumatic stress disorder (PTSD) increases the risk of suicidal ideation, suicide attempts, and suicide death (Holliday et al., 2020). As of 2020, veterans are at up to 6 times greater risk for suicide than nonveterans (Morral et al., 2023). Within military populations, PTSD is a critical factor to account for when attempting to ameliorate this elevated risk (Holliday et al., 2020). Although rates vary widely depending on study methodology, a recent review indicated that among U.S. military personnel or veterans with PTSD, the lifetime prevalence of suicidal ideation, suicide attempts, and suicide death is 35%, 20%, and 0.16%, respectively (Holliday et al., 2020). While mechanisms have not been conclusively identified, altered arousal, sleep disturbances, co-occurring mental health disorders (e.g., substance use and depression), and combat exposure with subsequent guilt all increase suicide risk for military populations with PTSD (Brown, Chen, et al., 2020; Bryan et al., 2013; Na et al., 2021; Orak et al., 2023; Vest et al., 2021; Weber et al., 2020). Importantly, emotion regulation and cognitive inflexibility have also been identified as critical treatment targets for reducing the risk of suicide among U.S. military personnel with PTSD (Bryan & Rozek, 2018; Rozek et al., 2022).

Prolonged exposure therapy (PE; Foa et al., 2007, 2019) is a frontline treatment for PTSD (e.g., American Psychological Association, 2025). The two critical components of PE include imaginal exposure to the memory of the index trauma and processing and in vivo exposure to real-world reminders of the trauma (Foa et al., 2007). PE also includes psychoeducation and breathing retraining (Foa et al., 2007). PE significantly reduces suicidal ideation in civilians (Brown, Belli, et al., 2020; Brown et al., 2018), veterans (Cox et al., 2016), and active-duty military personnel (Brown, McLean, et al., 2019). Reductions in PTSD symptoms and depression are key drivers of the reduction in suicidal ideation in PE (Brown, Zang, et al., 2019).

However, to our knowledge all published clinical trials evaluating patients with PTSD intentionally excluded patients who were deemed to be at very high risk for suicide. This exclusion criterion was usually implemented to ensure that participants would not be facing an acute suicidal crisis during PTSD treatment that would disrupt the course of care. As a result of this exclusion criterion, the safety of PE (without substantial modification or preparatory work, described below) among patients who are at higher risk for suicide has not been established. The high rates of suicidal ideation and attempts among patients with PTSD necessitate a treatment option that is safe, feasible, and effective for patients with PTSD who are thus at high risk of suicide.

The integration of dialectical behavior therapy (DBT; Linehan, 1987) with PE (DBT-PE; Harned et al., 2014) offers a useful treatment approach among patients with PTSD and has been used for those who also meet criteria for borderline personality disorder. A pilot evaluation of the DBT-PE protocol demonstrated that it was associated with lower rates of suicide attempts and self-harm compared to DBT alone among patients with PTSD and borderline personality disorder (Harned et al., 2014). Furthermore, a pilot effectiveness trial demonstrated that DBT-PE was associated with significantly greater reductions in PTSD symptoms than DBT alone and that the protocol was safe and tolerable (Harned et al., 2021). However, not all patients with PTSD meet the criteria for borderline personality disorder, and for those who do not, it might be unnecessary to wait for 2 months of DBT therapy (used to establish safety from life-threatening behaviors) before initiation of the trauma-focused part of therapy (PE). For those patients, there are no published safe and efficacious treatment options to guide their decision-making.

Brief suicide-focused interventions are an obvious candidate for integration with PE among patients who are reporting acute suicidal thoughts but who do not need (or cannot access) DBT. One such brief intervention is the Coping Long-Term with Active Suicide Program (CLASP; Miller et al., 2016, 2017). CLASP was originally conceived to support discharge for patients who had been psychiatrically hospitalized for suicidal thoughts and attempts. In its original design, CLASP included three individual sessions with the patient and therapists and one family session with a support person prior to discharge from the hospital. Upon discharge, the CLASP advisor had a series of check-in phone calls with the patient and their support person. The goals of CLASP include values clarification and goal setting; improving communication around suicidal crises between the patient and their support person; collaborative development of a safety plan and a Life Plan to support improved safety and build hope for the future, which includes a culmination of prior discussions about values and goals as an opportunity to great greater values-action consistency; and support in adhering to other treatments as needed for the patient. In a pilot randomized trial of CLASP vs. an enhanced assessment and follow-up control condition among patients who had been hospitalized for acute suicide risk, CLASP was associated with 44% fewer suicide attempts than control in the follow-up period (Miller et al., 2016). Among patients who presented to an emergency department for acute suicide risk, CLASP was associated with a 20% reduction in suicide attempts versus treatment as usual (Miller et al., 2017).

The therapeutic targets of CLASP are well-aligned with PE; for instance, in vivo exposures in PE can be tied to a patient’s larger life goals and value system. Likewise, PE may include a session that provides psychoeducation to a support person and garners additional support for the patient. Family members sometimes report being unclear on how to help their loved one while they complete PE, despite a desire to do so. When patients are worried about overengagement (i.e., becoming extremely upset in imaginal exposures to the point of not being able to learn) or about their ability to contain distress during or after imaginal exposure, therapists sometimes make a plan to ensure that imaginal exposures are not overwhelming, and the generation of a safety plan and Life Plan can be helpful in this regard. There are numerous points of synergy between CLASP and PE that have yet to be optimized or evaluated to date. Other brief evidence-based practices could be integrated with PE, including the Safety Planning Intervention (Stanley & Brown, 2012) or Crisis Response Planning (Bryan et al., 2017), but CLASP was chosen because of these numerous points of synergy with PE. It also offers a middle ground between one-contact interventions and more in-depth therapeutic approaches (Miller et al., 2022).

When a patient presents for treatment for PTSD, an obvious goal is to ensure that the patient receives treatment for their chief complaint. This is important because patients with PTSD have strong preferences for their treatment, and many prefer to do exposure therapy when asked (Becker et al., 2007; Schwartzkopff et al., 2021; Tarrier et al., 2006). When patients are turned away from trauma-focused therapies because of suicide risk, they might become discouraged from treatment-seeking. Being turned away from care at one site could lead to restarting the process of getting connected to care, which took, on average, 7–9 weeks before the COVID-19 pandemic and 12 weeks after (Peipert et al., 2022; Reichert & Jacobs, 2018). Turning a patient away based on suicide risk might also impair trust in the patient about the treatment process, especially when providers are reluctant to work with suicidal patients (Almaliah-Rauscher et al., 2020; Gvion et al., 2021) and a patient might have experienced a referral out after disclosing suicide risk before they present for care again. The CLASP-PE protocol offers a potential way to address this dilemma. When a patient with PTSD desires trauma-focused treatment, the CLASP-PE protocol may support the patient in attending to suicide risk concerns while also addressing the chief complaint.

The purpose of this pilot open-trial was to demonstrate proof-of-concept of the first integration of PE and CLASP, CLASP-PE. We hypothesized that CLASP-PE would be deemed feasible and acceptable based on the completion rate and qualitative feedback of the treatment protocol. We also hypothesized that CLASP-PE would be efficacious in terms of significant reductions in PTSD symptoms, suicidal ideation, and depression and would be safe in terms of low rates of suicide attempts. Lastly, based on previous research on the patterns of change in suicidal ideation in PE alone, we hypothesized that improvements in PTSD symptoms and depression would predict improvements in suicidal ideation in the treatment.

## Methods

### Participants

Participants (*N* = 14) were adult veterans or active-duty service members who met the criteria for PTSD according to the Clinician-Administered PTSD Scale for DSM-5 (Weathers, Blake, et al., 2013) and reported suicidal ideation with a plan, intent, or history of attempts. Participants were recruited by referral to the study by our partner veteran and Department of Defense (DoD) providers and via self-referral in response to brochures in the waiting rooms of providers. Participants had to have the ability to read and write in English and could not have a current psychotic or manic episode at the time of enrollment, a severe substance use disorder, or receiving a current alternative evidence-based PTSD or suicide-focused therapy (i.e., PE, CPT, CLASP, etc.). Most participants identified as male (85.7%) and White (57.1%), with ages ranging from 22 to 48 years old. Forty-three percent of the participants reported being separated or divorced, 53.8 partially completed college, and 42.9% were unemployed. Also, all participants who reported their service history endorsed having served in the two most recent military operations (Operation Enduring Freedom or Operation Iraqi Freedom), with the majority being veterans (84.6%) from the Army branch of service (69.2%) who have a service-connected disability for PTSD (71.4%). Two participants did not complete any assessments after the baseline assessment. Half of the participants (*n* = 7) included a support person in their treatment. When the research team learned about adverse events, they were reported to the study coordinator for tracking.

### Measures

#### PTSD Checklist for DSM-5 (PCL-5)

The PTSD Checklist for DSM-5 (PCL-5) is a 20-item self-report measure used to assess the presence and severity of posttraumatic stress disorder (PTSD) symptoms as defined in the DSM-5 (Weathers, Litz, et al., 2013). Respondents rate each item from 0 (*not at all*) to 4 (*extremely*) to indicate the degree to which they have been bothered by that symptom over the past month. The total score ranges from 0 to 80, with higher scores indicating greater severity of PTSD symptoms. The PCL-5 has demonstrated strong internal consistency (α = .94 to .96), test-retest reliability (rs = .74 to .85), and convergent and discriminant validity (Blevins et al., 2015; Bovin et al., 2016). This measure was administered at baseline, each treatment session, and at posttreatment.

#### Beck Scale for Suicide Ideation (BSS)

The Beck Scale for Suicide Ideation (BSS) is a 21-item self-report instrument designed to measure the severity of suicidal ideation (Beck et al., 1979). The first 19 items assess suicidal thoughts and attitudes, while the last two items inquire about previous suicide attempts. Each item is rated on a 3-point scale from 0 to 2, with total scores ranging from 0 to 38, where higher scores indicate more severe suicidal ideation. The BSS has demonstrated strong internal consistency, with Cronbach’s alpha coefficients ranging from .89 to .97 (Beck et al., 1988). This measure was administered at baseline, each treatment session, and at posttreatment.

#### Columbia-Suicide Severity Rating Scale (C-SSRS)

The Columbia-Suicide Severity Rating Scale (C-SSRS) is a semistructured interview used to assess the severity and intensity of suicidal ideation and behavior (Posner et al., 2011). The C-SSRS evaluates a range of suicidal ideation and behavior dimensions, including the frequency, duration, controllability, deterrents, and reasons for suicidal ideation, as well as the lethality and potential intent of any suicide attempts. The C-SSRS has demonstrated excellent inter-rater reliability (κ = .89 for ideation and κ = .94 for behavior) and strong internal consistency for ideation severity (Cronbach’s alpha = .73 to .92) (Posner et al., 2011). This measure was administered at baseline and at posttreatment.

#### Beck Depression Inventory (BDI-II)

The Beck Depression Inventory (BDI-II) is a widely used 21-item self-report inventory that assesses the severity of depressive symptoms (Beck et al., 1961). Each item represents a symptom of depression (e.g., sadness, pessimism, loss of pleasure) and is rated on a 4-point scale from 0 to 3, with total scores ranging from 0 to 63. Scores are interpreted as indicating minimal, mild, moderate, or severe depression. The BDI-II has demonstrated high internal consistency, with Cronbach’s alpha coefficients ranging from .81 to .94 in various populations (Beck et al., 1988). This measure was administered at baseline, each treatment session, and at posttreatment.

#### CLASP-PE

CLASP-PE was offered in a 13–20 twice-weekly session protocol (with length determined by clinical need) based on the protocols for CLASP and PE. Each clinical contact included safety planning, self-report measure completion, and homework review/assignment. Therapy sessions were 90 min, and a phone call check-in was scheduled between sessions to reinforce homework adherence and facilitate safety planning. Session 1 included psychoeducation about the program, gathering a detailed history of suicidal thinking and behaviors and trauma-related symptoms, and breathing retraining. The therapist discusses why the treatment directly targets PTSD symptoms concurrently with suicide risk, including that for some survivors of trauma, PTSD symptoms contribute to a desire to die. The therapist shares that by directly targeting PTSD symptoms while carefully monitoring and addressing suicide risk, patients may improve faster than in other treatment approaches, which only address PTSD symptoms or suicide risk in isolation. The therapist reviews the two strategies to attempt to address avoidance and trauma-related distress, namely in vivo exposure (or exposure to real-world people, places, or situations that cause distress) and imaginal exposure (or exposure to the memory of the trauma itself).

Session 2 included psychoeducation and discussion about common reactions to trauma, including suicidal thoughts and behaviors, values clarification, in vivo exposure rationale, and hierarchy generation with emphasis on content from values clarification. Session 3 included the development of a Life Plan consistent with the prior values-clarification and in vivo exposure discussion, as well as a meeting with a supportive significant other (as available) to share the Life Plan, the safety plan, and to facilitate communication and problem solving with the patient. After Session 3, weekly phone contacts with the supportive significant other were offered to provide feedback and information to help support the patient and provide continued problem-solving around clinical matters. Session 4 included the rationale for imaginal exposure and first imaginal exposure and processing of the index trauma. Safety checks occurred prior to and immediately after the first imaginal exposure, and participants were assigned to listen to an audio recording of the imaginal exposure and an in vivo exposure for daily homework. Sessions 5 until the second-to-last session included continuing with imaginal exposures and processing, switching to hot spots of the index trauma to reduce avoidance of discussing the trauma around Session 7, and continuing to update the safety plan as the patient progressed in treatment. The final session occurred between Sessions 10–16 based on clinical need and included one final imaginal exposure and processing, a final review of the safety plan, a review of progress and suggestions for future practice, and termination. Of participants who had at least one session, the average number of sessions was 10. Following treatment termination, patients had up to 3 check-in phone calls on an increasingly tapered scale up to 6 months after treatment termination.

#### Study Therapists

Study clinicians were PE-certified by the CTSA at the [redacted]. Clinicians received individual weekly supervision on CLASP-PE by [redacted]. Therapists completed an 8-hour training in safety planning and suicide management, consistent with the protocol for training CLASP clinicians in prior clinical trials. Therapists were provided with readings about suicide risk, assessment, and intervention, discussion and review of the CLASP-specific intervention targets, and didactic/one-to-one presentations of the CLASP model.

### Data Analysis Plan

Qualitative feedback for acceptability is reported in Table 1 and summarized below. Linear mixed-effects models were used to evaluate the change over time in PTSD symptoms, suicidal ideation, and depression symptoms in Stata v. 17. These models included a random intercept and random slope and evaluated the effect of time on the dependent variable. The slopes and intercepts of PTSD symptoms and depression symptoms were extracted for entry as a predictor of the change over time in suicidal ideation, with the Slope × Time effects evaluated after controlling for the random intercept of the dependent variable and the moderator (i.e., PCL-5 or BDI-II).

## Results

### Recruitment and Retention

A total of 21 participants were approached to participate, and 16 completed the informed consent. Two participants were lost to follow-up after completing the informed consent and did not provide any data for this manuscript, resulting in 14 participants in this sample. Two participants provided only baseline data. Of the 12 remaining participants, 8 participants (67%) completed all their sessions. Of the remaining 4 participants, 1 participant completed six sessions, 1 completed four sessions, 1 completed three sessions, and 1 completed two sessions.

### Clinical Characteristics

According to their Columbia-Suicide Severity Rating Scale (C-SSRS) interview at baseline, *n* = 11 reported a lifetime history of actual suicide attempts, and *n* = 2 reported an actual suicide attempt in the past 3 months. Based on their baseline Clinician-Administered PTSD Scale for DSM-5 (CAPS-5) interview, all the participants met the criteria for PTSD and reported past-month moderate to severe clinical levels of PTSD severity, with a median score = 38 (range: 22–53). In addition to PTSD, this sample also reported prior diagnoses of bipolar disorder (*n* = 3), alcohol use disorder (*n* = 1), and psychotic disorder (*n* = 1). Other comorbid diagnoses were not assessed as they were not pertinent to eligibility determination.

### Qualitative Feedback

Of the 8 participants who completed the program, 7 provided qualitative feedback (see Table 1). The feedback was generally very positive, with participants reporting that the imaginal followed by processing and in vivo exposures and breathing retraining (all traditional PE protocol elements) were helpful. Specifically, participants reported that it was helpful to revisit the memory of the trauma in that it either allowed them to “let go of the feelings,” (i.e., to no longer feel paralyzed by the memory/perpetrator of the trauma), or to come to new realizations about their autonomy. The only constructive feedback noted was about a need for clarity about some components in the therapist vs. the patient binders, which we will correct in future implementations. In addition, one participant suggested the possibility of involving multiple support people in case one becomes unavailable, which will be consistently communicated to individuals as a possibility in future trials.

### Change Over Time in PTSD (PCL-5)

Across all participants who had any PCL-5 data following baseline (*n* = 12), the mean reduction in PCL-5 scores was 40.7%. Of the 12 participants who had any data after baseline, 10 (83.3%) had a reduction in PCL-5 severity (range of reduction from 1.4%–95.8%), and 2 participants (18.2%) had an increase in PCL-5 severity (range of increase from 10.2–21.5%). One of the participants who reported an increase in PCL-5 severity provided very positive qualitative feedback about the program and fully completed it, whereas the other participant dropped out after Session 2 (see Table 1). All other participants who had data after baseline (*n* = 10) reported a decrease in PCL-5 scores regardless of whether they completed the program.

The intraclass correlation for the PCL-5 was .68, supporting the inclusion of a random intercept in the model, and a likelihood-ratio test confirmed that a random slope also significant improved model fit (X^2^ = 105.57, *p* < .001). An unstructured covariance matrix provided the best fit to the model. There was a significant reduction in PCL-5 over time (see Table 2).

### Change Over Time in Suicidal Ideation (BSS)

The intraclass correlation for the BSS was .84, supporting the inclusion of a random intercept in the model, and a likelihood-ratio test confirmed that a random slope also significant improved model fit (X^2^ = 16.76, *p* < .001). An unstructured covariance matrix provided the best fit to the model. There was a significant reduction in BSS over time (see Table 2).

### Change Over Time in Depression (BDI-II)

The intraclass correlation for the BDI-II was .75, supporting the inclusion of a random intercept in the model, and a likelihood-ratio test confirmed that a random slope also significant improved model fit (X^2^ = 76. 19, *p* < .001). An unstructured covariance matrix provided the best fit to the model. There was a significant reduction in BDI-II over time (see Table 2).

### Change Over Time in PTSD and Depression Predicting Change Over Time in Suicidal Ideation

There was a significant Time × PCL-5 slope interaction on BSS (coefficient = 0.200, 95% CI 0.116, 0.283, SE 0.043, z = 4.69, *p* < .001), controlling for the intercept of PCL-5, suggesting the change over time in PCL-5 significantly predicted the change overtime in BSS. Participants who had a steeper decline in PCL-5 scores had a steeper decline in BSS in treatment. This effect held after controlling for the slope and intercept of BDI-II. Likewise, there was a significant Time × BDI-II slope interaction on BSS (coefficient = 0.431, 95% CI 0.276, 0.586, SE 0.079, z = 5.45, *p* < .001), such that the change over time in BDI-II significantly predicted the change overtime in BSS.

### Rates of Suicide Attempts and Adverse Events

At baseline, 92% of participants reported a lifetime suicide attempt history on the C-SSRS; no actual, aborted, or interrupted suicide attempts or nonsuicidal self-injurious behaviors were reported over the follow-up period. There was one adverse event related to potentially life-threatening risk-taking behaviors associated with alcohol use during the follow-up period that was considered partially related to their participation in the treatment protocol. This event was not characterized by suicidal ideation or intent. No other adverse events or serious adverse events were reported.

## Case Vignette

Herein, we describe the details of a veteran (“John”) who completed the CLASP-PE treatment protocol as part of the study. We altered several details throughout to protect anonymity. John, a male veteran in his early 30s, presented with financial stress, job stress, household stress, gaining significant weight during the past year, no energy, panic attacks, and sleep difficulty (tossing and turning, sweating, nightmares). The veteran was married with a young child and worked in manual labor. He reported being easily frustrated and overwhelmed, anxious, and hypervigilant. The veteran was also experiencing relationship instability due to his partner’s infidelity, who reported plans to leave the home with their young child. This relationship instability prompted the veteran to call the VA suicide crisis hotline twice, and he was hospitalized for suicidal ideation for about 8 days. The veteran reported that prior to the relationship crisis, he had never experienced suicidal ideation. The veteran described no history of psychiatric issues until 2 months prior to starting therapy and had no history of a substance use disorder. He had started medications about 6 weeks prior to starting therapy. The participant received a prescription for an antidepressant (fluoxetine), nightmares (prazosin), and an as-needed anxiety medication (hydroxyzine).

Upon psychodiagnostic assessment, John met criteria for PTSD and major depressive disorder. The veteran had served as an active-duty service member for 10 years and had deployed to Afghanistan. While deployed, he reported experiencing mortar attacks almost daily. He also reported being bothered by memories of a “gruesome” deceased body of a fellow service member. Later in therapy, he reported having been physically attacked by a foreign national, resulting in the death of the foreign national in self-defense.

At baseline, John’s score on the PCL-5 was 48, and on the Beck Depression Inventory, it was 32. He reported thinking about suicide many times each day (lasting about 1 hour at a time) and reported a method in mind (to die by vehicle or by knife), a plan, and an intention to act on that plan prior to his recent hospitalization. He denied any history of suicide attempts.

### Course of Treatment

#### Session 1

The therapist asked John to share his experiences about the relationship between his PTSD symptoms and suicide risk, and he agreed that to him, it makes sense to try to address both symptoms simultaneously. The therapist shared that in the CLASP-PE program, the therapist will teach John skills to address suicide risk, such as a safety plan and building an overall Life Plan, as well as working on problem-solving skills to work through suicidal crises when they emerge. After psychoeducation, the therapist and John conducted an abbreviated Trauma History Interview to clarify the index trauma that would be the focus of imaginal exposures, which in John’s case was an incident involving serving on a security detail and being positioned to almost having to shoot someone in the head when they did not follow his command. The CLASP-PE version of the interview included an in-depth discussion of a suicide narrative. The therapist gathered information about the precipitants of his suicidal ideation, which included the relationship conflict with his wife, leading to her threatening to leave the relationship. The therapist asked about warning signs, coping strategies, and his process of ultimately getting to the hospital and recovering from the crisis.

The therapist then used the information from the suicide narrative to inform some possibilities for additions to John’s safety plan. John reported already having made a safety plan in the hospital, so the therapist and John reviewed the safety plan while engaging in a discussion about which aspects of the plan are likely/unlikely to be utilized and to help, which aspects should be changed, and whether John should add anything to his plan.

After a discussion of the safety plan, the CLASP-PE manual encourages a conversation about things that the therapist can do in session to help reduce the emotional intensity if needed. After reviewing some things that John felt would help him to calm down, which the therapist can refer to after imaginal exposure has been introduced, the therapist then introduced breathing retraining. In preparation for Session 2, the therapist shared a list of common values and asked for the patient to review that list prior to coming to the next session to facilitate a values clarification discussion.

Finally, the therapist revisited a conversation about the involvement of a support person in the protocol. Due to relational conflict and infidelity in his marriage, the patient decided that it would be best to involve his parents in the treatment. The therapist encouraged John to introduce this idea to his parents and see whether they would be willing to join in.

##### Phone-call check-in after Session 1.

During the first check-in phone call, the therapist began by assessing John’s physical location (in the event of an emergency). Then, the therapist assessed suicide risk for 5–10 min. John denied any suicidal ideation since the last meeting. After this assessment, they engaged in a discussion about the implementation of the safety plan. John confirmed access to his Safety Plan but denied a need to use it since he had not experienced suicidal ideation. Then, the therapist assessed homework completion, and John discussed progress on the assigned CLASP-PE homework. He reported continuing to struggle with his typical PTSD systems this week.

#### Session 2

John’s PCL-5 score remained at 48, the BDI-II was 31, and the BSS was 0. The therapist discussed the implementation of the safety plan. John reported that he did not need to make any changes to his safety plan. The therapist discussed additional ways for a veteran to keep his environment safe, including adding a picture of the area where he safely stored his knives/swords to serve as a reminder of his reason to live. John reported passive suicidal thoughts (“It would be easier if I did not wake up”) since the check-in phone call but reported that he was able to successfully use his support network to navigate the passive suicidal thoughts.

After reviewing homework, the therapist reviewed the Common Reactions to Trauma following the traditional procedures from the PE manual. The therapist discussed suicidal thoughts and attempts as a common reaction to trauma. The therapist showed the common reactions to trauma handout to John and asked him to review it for homework this week. Then, the therapist engaged John in a conversation about values clarification. She shared examples of common values, clarified the difference between goals and values, and then asked John to start reflecting on some of his big-picture values (see Appendix I with information changed to protect anonymity). For each value, John was encouraged to brainstorm a short-term, middle-term, and long-term goal connected to the value. He was then asked to further refine this list for homework.

Finally, the therapist discussed the rationale for in vivo exposure exercises, mentioning that avoidance maintains both PTSD symptoms and suicidal thoughts. The therapist supported John in building a hierarchy following the PE protocol, with each item linked to a value. John planned to go to public spaces and reduce safety behaviors while in those spaces for in vivo exposure (e.g., not scanning the environment, not backing his vehicle into a parking spot to allow for a faster exit, allowing himself to sit with his back to the door, etc.). John was reminded about bringing his parents to Session 3.

##### Phone-call check-in after Session 2.

John reported making progress toward his in vivo exposures of being in public while reducing safety behaviors and denied any issues with them at this point. He endorsed sleep difficulties and hypervigilance in the home. John discussed some problems with choosing how to prioritize values and goals and the therapist suggested strategies to support him in this effort.

#### Session 3

John’s parents were unexpectedly unable to join the session and rescheduled to join on Session 4. John’s scores on the PCL-5, BDI-II and BSS were stable (48, 31, and 0, respectively). John denied any suicidal ideation this week, and therefore did not need to use his Safety Plan, but he confirmed that he added pictures in the area where his swords are stored to serve as a reminder of his reason to live.

He worked on continuing values clarification by building a Life Plan document (see Appendix II), the goal of which was to integrate them into a more comprehensive vision for John’s future. The Life Plan includes space to document progress to help John achieve consistency between values and action. The therapist discussed how *in vivo* exposures are tied directly to Life Plan goals, and how imaginal exposures will reduce distress to make working toward goals easier.

##### Phone-call check-in after Session 3.

The veteran denied any suicidal ideation since the last session. He stated that his in vivo exposures were also going well related to decreasing safety behaviors in public places. He confirmed that his parents were planning to attend Session 4.

#### Session 4

John’s PCL-5 decreased to 30, his BDI-II decreased to 23, and his BSI remained 0. His parents joined the session over telehealth. The therapist reviewed the goals of the session, namely to discuss what John has been working on in treatment, to gather information about his parents’ concerns, and to plan to support John as he completed the treatment. The therapist invited John to share his experience of his PTSD symptoms and suicide risk with his parents in his own words. The therapist assisted in providing brief psychoeducation about suicide risk factors as well as PTSD symptoms and how they influence suicidal thinking. John’s parents reported that they were concerned primarily about his risk escalating in the context of ongoing marital strain. John was invited to review his safety plan with his parents, and they collaborated on planning for safety in the future. Then, John shared his Life Plan with his parents, including mentioning the big picture values and in vivo exposures that he is working on in the service of those values. The group discussed strategies to provide mutual support to each other throughout John’s treatment. The therapist shared that they would be in regular contact with the parents through check-in phone calls to invite them to be involved in treatment planning, as would be helpful to John. John’s parents left the session, and the therapist and John continued.

As John had denied any suicide risk, the therapist briefly checked in on the Safety Plan, which he reported did not need any changes this week. After the homework review, the therapist presented the rationale for imaginal exposure. Then, John began his first imaginal exposure related to processing his comrade, who was seriously injured and ultimately died. The pre-suicide urge was 0, and subjective units of distress (SUDS; Wolpe, 1969) at 5–20 min were 30, 55, 80, 30, and 40. The postsuicide urge was 0. Following the imaginal exposure, the therapist helped John engage in processing, where they discussed the cognitive and emotional impact of the trauma on him today. After processing, the therapist assessed safety and distress tolerance planning.

##### Phone-call check-in after Session 4.

John reported increased symptom distress due to a situation with his spouse. He discussed utilizing his Safety Plan for support and plans for safety throughout the weekend. John denied any suicidal ideation (SI) plan or intent.

#### Session 5

John’s PCL-5 decreased to 21, his BDI-II decreased to 13, and his BSS remained 0. He reported that he had not completed some of his homework because his wife was hospitalized, and he needed to stabilize the family situation. The therapist discussed the veteran’s current Safety Plan. He denied any SI since the last session.

John then completed imaginal exposure in which he revisited the memory twice. His presuicide urge was 0, and pre-SUDS was 35. His peak SUDS during the imaginal exposure was 50, and his post-SUDS was 40. His postsuicide urge was 0. Themes in processing focused on the surprise of what happened to his friend. After processing, the therapist and John agreed to continue in vivo exposures focused on going to public locations while reducing safety behaviors while adding on the practice of being a passenger in a car while dropping safety behaviors.

##### Phone call check-in after Session 5.

John reported no SI and discussed progress and roadblocks related to invivo exposures. He listened to the audio recording of the last session, which he reported went well. Separately, the therapist had a call with John’s parents. His parents discussed strategies to provide support for John as he is struggling with increasing stress at home. The therapist discussed some strategies to try to provide support to John based on what they had discussed in their earlier family session. The parents were amenable to these ideas.

#### Session 6

On his self-report questionnaires, John’s PCL-5 score increased to 27, his BDI-II decreased to 9, and his BSS score remained at 0. He completed his in vivo exposures related to being a passenger in a car while dropping safety behaviors and being in public places without his safety behaviors. He also reported having reviewed the Life Plan and made some adjustments to it due to some changes in his home life due to his marital strain.

During the imaginal exposure, John completed the memory 1.5 times. His presuicide urge was at 0, and his pre-SUDS was 25. His peak and post-SUDS was 40. His postsuicide urge was 0. In processing, the patient revealed that it might be sensible to switch to another type of trauma memory, which has been haunting him more recently (focused on having to kill an insurgent in self-defense). The therapist and patient discussed this at length in terms of the pros and cons versus continuing to process the original memory, as it is not typical to switch memories in PE. John reported that he was feeling much more bothered by this alternative trauma and feeling increased confidence about his ability to process it in therapy, which has previously discouraged him from focusing on the alternative trauma. The therapist and John planned to switch to that memory during the next session after giving John an opportunity to continue listening to the imaginal exposure audio recordings this week.

##### Phone call check-in after Session 6.

John reported that he has not had SI at all and feels “much lighter” lately. He reported continuing to maintain frequent interaction with support persons and noticed progress with his symptoms. He listened to his audio session once since the session and talked about his plan to conduct in vivo exposures over the weekend.

#### Session 7

On the self-report measures, John’s score on the PCL-5 decreased to 9, the BDI-II decreased to 4, and the BSS remained 0. As John continued to deny SI, the session content shifted to the new trauma for imaginal exposure. This trauma related to John being physically attacked while on base, resulting in the death of someone else. His presuicide urge was 0, pre-SUDs was 35, peak and post-SUDS was 75, and the postsuicide urge was 0. Themes in processing related to guilt about having to kill this person and regretting that he had been put in that situation. In processing, John also talked about dissociation, which he observed as a coping mechanism for the trauma memory. He also discussed his bodily reactions during the trauma and some trauma-related cognitions that were generated for him because of the trauma.

##### Phone call check-in after Session 7.

John denied SI and reported feeling and doing well and that he was making progress toward his homework goals. Separately, the therapist also spoke with John’s parents about their desire to set boundaries with John and safely navigating emotional crises. The therapist offered validation and some suggestions, which the parents were willing to try.

#### Session 8

On self-report measures, John’s PCL-5 increased to 21, and his BDI-II increased slightly to 10, whereas his BSS remained at 0. John denied any SI since the last session, stated that he feels “the opposite,” and he noted a strong desire to be around to help others. Since his suicide risk remained stable, John proceeded directly to imaginal exposure. His presuicide urge was 0, pre-SUDS was 35, peak SUDS was 85, and post-SUDS was 85. His postsuicide urge was 0. Processing continued to focus on trauma-related cognitions on themes of guilt, in particular survivors’ guilt. Processing also included a discussion of his physical body reactions during imaginal exposure and the trauma itself, visual reminders, and new pieces of the memory that he had not previously discussed.

##### Phone call check-in after Session 8.

John denied suicidal ideation since the last session. He reported doing well and discussed plans for upcoming in vivo exposures.

#### Session 9

On self-report measures, John’s PCL-5 score decreased to 14, his BDI-II was 9, and his BSS remained at 0. The only notable characteristic of the session was that the therapist suggested that John switch to hot spots for the imaginal exposure this week, consistent with the PE protocol. John was amenable to this plan and opted to focus on the part of the memory when he was being attacked. His presuicide urge was 0, pre-SUDS was 25, peak SUDS was 85, and post-SUDS was 85. His postsuicide urge was 0. Themes in processing focused on what it felt like to survive the attack, as well as his perceptions of contributions to the attack.

##### Phone call check-in after Session 9.

John denied SI since the last visit. Due to his busy work schedule, he did not engage in any in vivo exposure practices yet this week, but he discussed a plan to go to the zoo and reduce safety behaviors. Separately, the therapist had a support call with John’s father to discuss how John’s family can continue to provide him with support.

#### Session 10

On the self-report questionnaires, the PCL-5 decreased to 8, the BDI-II remained stable at 9, and the BSS remained at 0. The only notable feature of the session was during the Safety Plan review. Considering his increased marital tension, the therapist introduced the idea of imagining some possible high-risk situations that could be coming his way, and John discussed strategies to cope with those situations if they emerge. They discussed this in relation to the Safety Plan, but John felt that no changes to the Safety Plan were needed at this time.

During the imaginal exposure, the focus continued to be on hot spots. His presuicide urge was a 0, his pre-SUDS was a 25, his peak SUDS was 65, and his post-SUDS was 65. His postsuicide urge was 0. Themes in processing included a focus on how his SUDS were decreasing as he spent more time with this memory.

##### Phone call check-in after Session 10.

John reported that he has been working on his in vivo exposures and expressed positive feedback at his place of employment. He denied SI since the last meeting.

#### Sessions 11 and 12

On self-report measures, John’s PCL-5 decreased to 2, his BDI-II decreased to 1 (0 Session 12), and his BSS remained 0. In Session 11, during processing, John returned to a discussion about his body’s response to recalling the memory versus what he felt during the memory. He also discussed some of his cognitions about the trauma now and reported more balanced thinking about his previous guilty cognitions. He agreed to extend his in vivo homework to include listening to traumatic sounds that resemble the sounds of combat and gunshots. In Session 12, themes in processing focused on changes in this memory and on perceptions of other traumas from deployment. John felt more confident in being able to discuss other traumatic experiences without feeling overwhelmed with emotions.

##### Phone call check-in after Session 12.

John denied suicidal ideation. He discussed positive changes he has noticed while listening to his audio recordings of sessions, having a positive mood, and plans for more invivo exposures over the weekend.

#### Session 13: Final Session

For the final session, John’s PCL-5 was 2, BDI-II was 0, and BSS was 0. John denied any SI since the last session and reflected again on positive coping strategies that he has learned to help him cope with suicidal and other kinds of crises. Consistent with the protocol, he engaged in a final round of imaginal exposure, including the entire original trauma memory, which he went through once. His presuicide urge was 0, pre-SUDS was 10, peak SUDS was 20, post-SUDS was 20, and postsuicide urge was 0.

John reviewed his progress: His PCL-5 decreased from 48 to 2, and his BDI-II decreased from 32 to 0 over the course of treatment. After his hospitalization, which prompted participation in the trial, he did not experience any exacerbations in suicide risk. He reflected on his positive use of coping mechanisms to encounter adversity and his ability to use his safety plan in future crises. John reported that he noticed a significant improvement in his functioning, which he attributed to the in vivo exposure practices. He noted that he was able to be in public with significantly decreased safety behaviors and has shown improvement in his vocational situation. Overall, John reported feeling very satisfied with his progress in the program. John reviewed his scores on his in vivo hierarchy from Session 2 and compared them to new ratings, noting marked decreases in all domains.

The therapist and John discussed the plan to have some follow-up check-in phone calls. The therapist thanked him for his participation in and dedication to the program and scheduled the check-in call.

In his follow-up interview with the study staff, John reported that he was “overjoyed” with how the study turned out for him and reported a “15” on a satisfaction scale ranging from 0 – 10. He reported learning a lot of tools to help him navigate crises and found that his therapist was extremely supportive and helpful.

##### Phone call check-in: 1-week follow-up call.

John reported that, generally, things were going well. He discussed anger related to foreign conflicts but felt good about his own general issues. He denied any suicidal ideation and expressed continued use of his support network. He described some psychosocial stressors but reported that he has been able to cope with them by utilizing grounding/mindfulness techniques and analyzing his choices. He and the therapist scheduled a check-in phone call for 3 weeks later.

##### Phone call check-in: 1-month follow-up call.

John reported that things were going well. He discussed methods to prevent reverting to avoidant behavior. He denied SI.

##### Phone call check-in: 2-month follow-up call.

John reported an increase in some of his symptoms but denied SI. He realized that he had stopped utilizing some of the skills that he had learned. He discussed how he has been coping with some new and prior stressors. He also discussed skills that he had previously learned and how he could apply those skills to his current situation.

## Discussion

This pilot study demonstrated that integrating PE with CLASP (CLASP-PE) was safe, acceptable, and feasible and had preliminary evidence to support efficacy. Participants who received CLASP-PE reported significant reductions in PTSD symptoms, suicidal ideation, and depression symptoms in an open-trial evaluation of the protocol. Furthermore, the degree of reduction in PTSD symptoms and depression symptoms predicted the degree of reduction in SI over time. No participants reported actual, aborted, or interrupted suicide attempts or self-injury during the trial.

Patients with PTSD commonly struggle with suicidal thoughts and behaviors, and there are no evidence-based clinical practice guidelines to simultaneously address suicidal thoughts and PTSD-related distress outside of intensive interventions like DBT-PE. This study is the first to demonstrate that PE can be successfully utilized with suicide prevention strategies borrowed from the CLASP program to provide trauma-focused care to patients with PTSD who are acutely struggling with suicidal thoughts. Several reports have demonstrated PE’s associating with a reduction in SI over the course of treatment (Brown, Belli, et al., 2020; Brown, McLean, et al., 2019; Cox et al., 2016). However, there may have been a floor effect regarding this benefit insofar as in these prior investigations acute suicide risk was used as an exclusion criterion. This study demonstrates that among patients with acute suicidal thinking and behaviors, CLASP-PE might serve as a safe and efficacious treatment option.

The CLASP-PE protocol might not be suitable for all patients with PTSD who have acute suicide risk. Some patients with borderline personality disorder and PTSD might benefit from the DBT-PE protocol (Harned et al., 2014, 2021), which has preliminary evidence for feasibility and acceptability and is more suitable for patients with this particular comorbidity. However, most patients with PTSD who report current suicidal ideation and behaviors do not meet criteria for borderline personality disorder. For those patients, the DBT-PE protocol might be too intensive or prolonged, and the CLASP-PE protocol might offer a viable alternative.

The CLASP-PE protocol targets symptoms of PTSD and suicidal thinking in tandem. While encouraging patients to engage in exposure to reduce trauma-related avoidance, the CLASP-PE protocol anchors exposure practice to big-picture values that are consistent with building a life worth living. Prior to offering the patient to share the story of their traumatic experience in imaginal exposure, it is important to build a safety plan with the patient to ensure that they feel supported in navigating suicidal crises that may emerge in the context of emotional provocation. The CLASP-PE protocol also encourages collaboration with a support person, where available, to improve communication about both suicide risk and PTSD symptoms and to brainstorm a plan to support the patient as they work through their trauma memories. Through phone call check-ins in the CLASP-PE protocol, the patient, their support person, and the therapist have an opportunity to troubleshoot when issues arise in the context of safety planning, in vivo exposure, imaginal exposure, or otherwise. These protocol adaptations require some reorganization of content in the original PE protocol but add only one additional session to the overall length of treatment. The study therapists, who were already trained as experts in PE, required one 8-hour training in the CLASP-PE protocol, and we anticipate that learning the CLASP augmentation to PE will not require a significant training burden for therapists, though this remains to be formally evaluated.

This pilot project has some important limitations that require consideration. First, this was a small sample comprised of mostly male participants, which might not generalize to the larger population. Second, the pilot trial did not include a comparison group, which will be a necessary next step in this program of research, and it did not include quantitative benchmarks for acceptability and feasibility beyond qualitative interviews. Third, some participants prematurely dropped out of the protocol. The reasons for dropout included treatment nonadherence (failure to respond to treatment scheduling and/or failing to attend several sessions in a row), inability to commit to treatment due to external life stressors, and the need for imminent inpatient care outside of research. No participants reported that they were dropping out of the study because of fears that the treatment was making their suicidal thinking or PTSD symptoms worse. While the protocol is less burdensome than other existing therapeutic approaches, some participants might find that the protocol is still too burdensome at 90 min in length per session. Fourth, this protocol was evaluated in a sample of military personnel and veterans, and the results might not generalize to a civilian sample. Finally, only half of the participants included a support person; this reflects a pattern of challenges with patient comfort/willingness to involve a support person in the larger suicide prevention literature. For instance, in one study in the VA, over 350 veterans were approached to participate, and 39 were randomized, with the majority deciding not to participate because of hesitancy in involving family members (Goodman et al., 2022). There is a need for advancements in the field to support patients in overcoming obstacles to involving third parties when clinically appropriate and safe.

In summary, this pilot study demonstrates the acceptability, feasibility, and preliminary efficacy of an integrated treatment protocol, CLASP-PE, to simultaneously address acute suicidal thinking and PTSD symptoms. In this open trial, participants reported significant improvements in PTSD symptoms, depression symptoms, and suicidal ideation. Furthermore, the degree of reduction in PTSD symptoms and depression symptoms predicted the degree of improvement in suicidal ideation in treatment. Future research should evaluate the efficacy of the intervention in a randomized controlled trial.

## Figures and Tables

**Table 1:** Qualitative Feedback

Overall reactions?	What did you find helpful?	What did you not find helpful?	What suggestions would you have?	Is there anything that you would like to share?	PCL-5 D pre-post-tx
Super excited, sleep is great, study gave me tools to help in case sleep starts to decline. Overjoyed at how the study turned out.	[My therapist] checked in to see if I was doing okay randomly. I had easy access to talk to her even on weekends. I learned a lot of tools with in vivo and putting down on paper with the binder and keeping it going every single day.	At first things in general did not feel like they were going to be helpful, but after time it was all helpful.	Nothing comes to the top of [my] head because it really worked for [me]. Maybe offer different hours of availability.	Yes, for any patients that are struggling if they really want to get better, they have to find the time to do it. Because putting in the work definitely works.	48–2
I liked it. It was good.	The focusing on the part of the event that was most bothersome-	Nothing	Can’t think of anything	Breathing techniques were really helpful. Therapist allowed to deal with issues that were bothering [me] that were more recent	56–14
Grateful for the study and it helped a lot, and not feeling paralyzed by perpetrator.	It gave me a strong foundation and was able to know that [I am] in control and that [I] am an adult now and it can’t happen to [me]	No not really. The therapy was very helpful.	Everything was good.	No, just appreciate that [I] could be a part of it.	65 −79
Overall, it was very helpful.	The therapist and treatment as a whole - it definitely was good. [My therapist] specifically did a good job of keeping me involved and grounded.	Disorganization and the notebook on mine and [the therapist’s] end. Random page missing.	Wished [I] had this in the army while I was still in to offer PE/therapy at the bases.	Not really	52–39
Being enmeshed in the memory to see why it was bothering [me]	Going back over one memory- the imaginals and to let go of the feelings	Nothing, it was all helpful.	None, it’s a wonderful program.	No, it’s great and [my therapist] was very helpful.	45–5
Painful. Doing the therapy was painful.	Having and learning better coping skills.	The breathing and the in vivo exposures/activities	Would have preferred to do therapy in person	No, not really.	50 –18
Beneficial, process was helpful. The imaginals & hearing myself on the recordings was helpful.	Felt it’s important to participate in each part to get the most out of it.	The patient and therapist binders did not match, but they were able to work through it.	Maybe to have 2 support people instead of 1 in case the one support person wasn’t available.	Telehealth and scheduling flexibility was great. I learned to deal better with my anxiety and PTSD symptoms are less. I have more patience now with my kids.	42–3

**Table 2:** Change in PTSD, BSS, and BDI-II Over Time

	Coefficient (95% CI)	SE	Z	p
**PCL-5**				
Time	−2.312 (−3.794, −0.830)	0.756	−3.06	< .01
Intercept	53.669 (48.360, 58.978)	2.709	19.81	<.001
**BSS**				
Time	−0.4131 (−0.785, −0.077)	0.180	−2.39	.017
Intercept	12.412 (8.103, 16.721)	2.198	5.65	<.001
**BDI-II**				
Linear Time	−1.621 (−2.404, −0.837)	0.400	−4.05	<.001
Intercept	32.747 (26.919, 38.575)	2.973	11.01	<.001

*Note*. PCL-5 = PTSD Checklist for DSM-5; BSS = Beck Scale for Suicide Ideation; BDI-II = Beck Depression Inventory-II

## Appendix I. High-Priority Values Examples

**Table:**

**Value 1:** Being a dedicated and loving family member	**Value 2:** Being a loyal friend
**Short-term goal:** Increase communication with family members; call and text them more frequently, at least once a week **Medium-term goal:** Have more vulnerable conversations with family members in the next month, to tell them how I’m really doing **Long-term goal:** See extended family in person 1 x/year	**Short-term goal:** Initiate more conversations with Bob & Bill **Medium-term goal:** Spend time outside of work with a colleague one time per quarter **Long-term goal:** Practicing going to public places to hang out 1:1 with a friend and being more open about what is going on with me
**Value 3:** Physical and mental fitness **Short-term goal:** Workout twice per day **Medium-term goal:** Pass my physical fitness test **Long-term goal:** Practice physical fitness in public spaces to get more used to being around other people	**Value 4:** Persistence in my goals **Short-term goal:** Complete a course that I started **Medium-term goal:** Get promoted to Captain **Long-term goal:** Follow-through on commitment to doing big obstacle course race
**Value 5:** Being supportive of others **Short-term goal:** Make sure to praise crew at least once per week **Medium-term goal:** Train crew to make sure that they have what they need for their roles **Long-term goal:** Develop a fitness program to support crew in passing their physical fitness test	**Value 6:** Connection with others **Short-term goal:** Remind myself that not everyone has the same values and work ethic as me, and to practice acceptance of their values and work ethic **Medium-term goal:** Practice calmly explaining why I am frustrated as it arises **Long-term goal:** Get to know my crew better and learn about their goals, and adjust my expectations accordingly

## Appendix II. CLASP-PE: Life Plan

**Table:**

VALUES (Your personal life direction: for example, health, family, friends, career, romantic)	GOALS (The things you want to achieve that are consistent with your values)	PROGRESS (How is it going?)
1-Being a dedicated and loving family member	See extended family in person 1 x/year Practicing going to public places to hang out 1:1 with a friend and being more open about what is going on with me Practice physical fitness in public spaces to get more used to being around other people	Session 2–4: Practicing being in public more while reducing safety behaviors Session 4: Started imaginal exposure Session 5–10: Practicing riding in a car without safety signals as a passenger Session 7: Switched imaginal exposure memory Session 9: Started hot spots of imaginal exposure Session 11: Exposure to loud sounds that remind me of the trauma (gunshots)
2-Being a loyal friend	Follow-through on commitment to doing big obstacle course race	
3-Physical and mental fitness	Develop a fitness program to support crew in passing their physical fitness test	
4-Persistence in my goals	Get to know my crew better and learn about their goals, and adjust my expectations accordingly **In vivo exposures that** correspond (# on hierarchy): Going to public spaces Being around loud noises	
5-Being supportive of others	Being a passenger (all while reducing safety behaviors)	
6-Connection with others	**Imaginal exposures that correspond:** Memory related to having to almost kill insurgent; memory related to having to kill insurgent	
